# Orexin-1 and orexin-2 receptor antagonists reduce ethanol self-administration in high-drinking rodent models

**DOI:** 10.3389/fnins.2014.00033

**Published:** 2014-02-25

**Authors:** Rachel I. Anderson, Howard C. Becker, Benjamin L. Adams, Cynthia D. Jesudason, Linda M. Rorick-Kehn

**Affiliations:** ^1^Medical University of South CarolinaCharleston, SC, USA; ^2^Charleston Alcohol Research CenterCharleston, SC, USA; ^3^Ralph H. Johnson VA Medical CenterUSA; ^4^Lilly Research Laboratories, Eli Lilly and CompanyIndianapolis, IN, USA

**Keywords:** hypocretins/orexins, ethanol consumption, operant progressive ratio, P rat, C57BL/6J mouse

## Abstract

To examine the role of orexin-1 and orexin-2 receptor activity on ethanol self-administration, compounds that differentially target orexin (OX) receptor subtypes were assessed in various self-administration paradigms using high-drinking rodent models. Effects of the OX_1_ antagonist SB334867, the OX_2_ antagonist LSN2424100, and the mixed OX_1/2_ antagonist almorexant (ACT-078573) on home cage ethanol consumption were tested in ethanol-preferring (P) rats using a 2-bottle choice procedure. In separate experiments, effects of SB334867, LSN2424100, and almorexant on operant ethanol self-administration were assessed in P rats maintained on a progressive ratio operant schedule of reinforcement. In a third series of experiments, SB334867, LSN2424100, and almorexant were administered to ethanol-preferring C57BL/6J mice to examine effects of OX receptor blockade on ethanol intake in a binge-like drinking (drinking-in-the-dark) model. In P rats with chronic home cage free-choice ethanol access, SB334867 and almorexant significantly reduced ethanol intake, but almorexant also reduced water intake, suggesting non-specific effects on consummatory behavior. In the progressive ratio operant experiments, LSN2424100 and almorexant reduced breakpoints and ethanol consumption in P rats, whereas the almorexant inactive enantiomer and SB334867 did not significantly affect the motivation to consume ethanol. As expected, vehicle-injected mice exhibited binge-like drinking patterns in the drinking-in-the-dark model. All three OX antagonists reduced both ethanol intake and resulting blood ethanol concentrations relative to vehicle-injected controls, but SB334867 and LSN2424100 also reduced sucrose consumption in a different cohort of mice, suggesting non-specific effects. Collectively, these results contribute to a growing body of evidence indicating that OX_1_ and OX_2_ receptor activity influences ethanol self-administration, although the effects may not be selective for ethanol consumption.

## Introduction

Orexins A and B are neuropeptides synthesized in neurons originating in the lateral hypothalamus (LH) that project throughout the brain and bind to two widely expressed G-protein coupled receptors, orexin-1 (OX_1_) and orexin-2 (OX_2_). OX_1_ receptors selectively bind orexin A, whereas OX_2_ receptors bind orexin A and B with equal affinity (Sakurai et al., 1998). This neuropeptide system plays an established role in numerous behavioral and regulatory functions including sleep, arousal, and feeding behavior (Willie et al., 2001; Sakurai, 2002). While orexin neurons in the dorsomedial hypothalamus are believed to regulate arousal and stress responses, orexin neurons within the LH are hypothesized to play a role in regulating reward processing for natural rewards as well as drugs of abuse (Harris and Aston-Jones, 2006). This has led to the suggestion that the orexin system is involved in addiction (for review, see Sharf et al., 2010; Mahler et al., 2012). Although evidence previously supported functional differences between the two receptors, with OX_2_ receptor activity more closely related to arousal and OX_1_ receptor activity more closely associated with reward (Aston-Jones et al., 2010), more recent research has revealed a role for OX_2_ receptors in reward processes as well (Shoblock et al., 2011; Brown et al., 2013).

While a growing body of literature has shown that the orexin system interacts with drug-seeking behavior induced by numerous drugs of abuse such as cocaine, nicotine, and opiates (reviewed by Mahler et al., 2012), the orexin system also has been implicated in the motivational properties of ethanol. Administration of orexin A into the paraventricular nucleus within the LH resulted in elevated ethanol consumption in Sprague-Dawley rats (Schneider et al., 2007). Orexin antagonists that target both OX_1_ and OX_2_ receptors have been shown to influence ethanol consumption (Kim et al., 2012). For example, systemic administration of the OX_1_ receptor antagonist SB334867 reduced ethanol intake and preference in Sprague-Dawley rats (Moorman and Aston-Jones, 2009). This same antagonist has been shown to reduce relapse drinking, operant responding, and both cue- and stress-induced reinstatement in other rat strains (Richards et al., 2008; Dhaher et al., 2010; Jupp et al., 2011). The OX_2_ receptor antagonist JNJ-10397049 also reduced ethanol self-administration and expression of ethanol conditioned place preference (Shoblock et al., 2011). Central administration of the OX_2_ receptor antagonist TCS-OX2-29 reduced ethanol intake but did not alter cue-induced reinstatement of responding for ethanol (Brown et al., 2013). Although the dual OX_1/2_ receptor antagonist almorexant has been shown to reduce operant ethanol self-administration when injected either systemically or directly into the ventral tegmental area (VTA; Srinivasan et al., 2012), effects of dual antagonism of both orexin receptors on ethanol consumption have not been as thoroughly explored.

There is some evidence to suggest that orexin antagonists may be particularly effective in subjects that show a high preference for ethanol. For example, an OX_1_ receptor antagonist was more effective in reducing ethanol consumption among outbred Sprague-Dawley rats that demonstrated high vs. low ethanol preference (Moorman and Aston-Jones, 2009). Further, several studies that reported orexin antagonist-induced reductions in ethanol self-administration used rats selectively bred for high ethanol preference (Lawrence et al., 2006; Dhaher et al., 2010; Jupp et al., 2011; Brown et al., 2013). Taken together, these results suggest that blocking orexin activity in the brain may be particularly effective in reducing ethanol consumption under conditions in which subjects exhibit a high propensity for ethanol self-administration. The present study was designed to characterize the relative contributions of OX_1_ and OX_2_ receptor-mediated signaling in modulating ethanol consumption using three different self-administration paradigms in high-drinking rodents. The OX_1_ receptor antagonist SB334867 (Smart et al., 2001) is >1000-fold selective for OX_1_ over OX_2_ receptors, whereas the novel OX_2_ receptor antagonist *N*-((1H-imidazol-2-yl)methyl)-*N*-([1,1′-biphenyl]-2-yl)-4-fluorobenzenesulfonamide hydrochloride (LSN2424100) is >200-fold selective for OX_2_ over OX_1_ receptors (Fitch et al., 2014). The dual OX_1_/OX_2_ antagonist almorexant (ACT-078573; Brisbare-Roch et al., 2007), which is approximately 1.3-fold OX_2_-preferring (Fitch et al., 2014), was also tested for comparison to the more selective compounds. Each of these compounds was tested in three different experiments: home cage free-choice drinking in female P rats, progressive ratio operant responding maintained by ethanol in female P rats, and ethanol consumption in a binge-drinking model (drinking-in-the-dark) in male C57BL/6J mice. Some of the P rat experiments included either the inactive enantiomer of almorexant as a negative control or naltrexone as a positive control.

## Materials and methods

### Subjects

All experiments were conducted in compliance with the Guide for the Care and Use of Laboratory Animals under protocols approved by the local Institutional Animal Care and Use Committees. Rat experiments were conducted in adult female selectively bred Alcohol-Preferring (P) rats generously supplied by the Indiana University School of Medicine (maintained as a private colony at Taconic Inc., Germantown, NY). For the home cage ethanol consumption studies, a total of 32 female P rats were individually housed with 24-h *ad libitum* access to 15% (v/v) ethanol, water, and food. All 32 P rats had chronic access to ethanol in the home cage for approximately 8–14 months before the current studies were conducted. P rats were divided into 3 groups. One group (*n* = 10) was used to test the effects of SB334867 and a second group (*n* = 11) was used to test the effects of LSN2424100 (one rat was excluded from the experiment due to low baseline drinking). A within-subjects experimental design was used to test the OX_1_ and OX_2_ receptor antagonists. These rats, along with another group of 11 (i.e., all 32 P rats) were tested in the almorexant study using a between-subjects design (*n* = 8/dose).

A separate cohort of female P rats (*n* = 10) used in operant experiments were pair-housed with food and water available *ad libitum* and maintained on a 12-h light/dark cycle (lights on at 6:00 AM). All operant procedures were conducted during the light phase (between 10 AM and 4 PM). In order to reduce the total number of animals used, within-subjects designs were employed for the operant and home cage consumption experiments. To avoid potential carryover effects, a 3–4 day washout period was imposed between different drug doses, and a 4–7 day washout period was included between the different drug experiments. Baseline performance of the rats (operant and home cage consumption) was monitored on non-dose days to confirm that ethanol intake returned to baseline levels prior to testing.

For the mouse experiments, a total of 166 adult male C57BL/6J mice (Jackson Laboratories, Bar Harbor, ME) were used in the binge drinking experiments, which were conducted using a between-subjects design. Mice were individually housed throughout experimentation under a 12-h reverse light/dark cycle (lights off at 8:00 AM). All testing occurred during the dark cycle.

### Drugs

*N*-((1H-imidazol-2-yl)methyl)-*N*-([1,1′-biphenyl]-2-yl)-4-fluorobenzenesulfonamide hydrochloride (LSN2424100), SB334867, (S)-almorexant (ACT-078573), and the inactive (R) enantiomer of almorexant were synthesized at Lilly Research Laboratories (Indianapolis, IN). Naltrexone hydrochloride was purchased from Sigma Aldrich (St. Louis, MO). For rat experiments, the OX_1_ antagonist SB334867 was dissolved in a vehicle of 10% (2-hydroxypropyl)-β-cyclodextrin, 2% dimethyl sulfoxide, and 0.05% lactic acid in water, and administered by intraperitoneal (i.p.) injection in a dose volume of 1 ml/kg. The OX_2_ antagonist LSN2424100 was suspended in 1% carboxymethyl cellulose, 0.25% polysorbate-80 and 0.05% Dow antifoam in water, and administered by i.p. injection in a dose volume of 1 ml/kg. The mixed OX_1/2_ antagonist almorexant, and its inactive enantiomer, were dissolved in a 20% Captisol solution and administered orally (p.o.) in a dose volume of 1 ml/kg. Naltrexone was dissolved in water with the addition of 15 μl 85% lactic acid.

For mouse experiments, SB334867 was dissolved using 0.01% polysorbate-80 in saline. Almorexant was dissolved in 20% Captisol in water. LSN2424100 was suspended using 1% carboxymethyl cellulose and 0.25% polysorbate-80 in water. All compounds were administered by i.p. injection at a dose volume of 10 ml/kg.

### Procedure

#### Home cage 2-bottle choice drinking in P rats

P rats were housed individually in TSE LabMaster cages (TSE Systems, Bad Homburg, Germany) with food, water, and 15% ethanol (v/v) available at all times. Water and ethanol intake (in ml) were measured once every 5 min throughout the 12-h dark cycle and recorded for later analysis. In the first experiment, rats (*n* = 10) received vehicle, 3, 10, or 30 mg/kg SB334867 (i.p.), 60 min before onset of the 12-h dark phase of the light-dark cycle, using a within-subjects design. In the second experiment, rats (*n* = 10) received vehicle, naltrexone (10 mg/kg), or LSN2424100 at doses of 10 or 30 mg/kg (i.p.), 60 min before onset of the 12-h dark phase, using a within-subjects design (one rat was excluded from the experiment due to low baseline drinking). In the third experiment, rats (*n* = 32) received vehicle, naltrexone (10 mg/kg), or S-almorexant at doses of 60 or 100 mg/kg (p.o.), 60 min before onset of the dark cycle, using a between-subjects design. Naltrexone was included in the study design as a positive control, since this dose of naltrexone has been shown to effectively reduce ethanol consumption in P rats under these testing conditions. For all experiments, a 60-min pre-treatment period was chosen so that the onset of the dark cycle roughly coincided with the time at which maximal brain concentrations were achieved (data not reported). Consumption of water and ethanol was measured during the first 3 h of the dark cycle, based on the short half-lives and high metabolism of the compounds.

#### Operant progressive ratio responding in P rats

P rats were trained and tested 5 days per week in standard rat operant chambers (Med Associates, St. Albans, VT), housed within sound attenuating boxes. Operant chambers measured 30.5 × 24.1 × 21 cm, with clear Plexiglas front and back walls, modular aluminum sidewalls, a metal bar floor and Plexiglas ceiling. A food cup was located in the center of one sidewall with retractable levers on either side of the food cup. A liquid dipper device allowed the delivery of 0.1 ml of 15% ethanol (v/v) into the food cup. A computer running the MED-IV software package (Med-Associates, St. Albans, VT) controlled stimulus presentations and recorded lever presses. Once subjects were trained to lever press for ethanol reinforcement on a fixed ratio-1 (FR1) schedule of reinforcement, the response requirement for each reinforcement was slowly increased to FR2 and then FR3 over 1–2 weeks. When rats demonstrated a stable level of responding on the FR3 schedule, progressive ratio testing began. The progressive ratio schedule involved increasing response requirements within each session. The response requirement increased from 1 to 2 after three ethanol presentations, and continued to increase by two after every three ethanol presentations (see Rodd et al., 2003). Experimental sessions terminated after 60 min. Total responses on the active and inactive levers, breakpoints [defined as the highest fixed ratio (FR) value reached during the session], and the amount of ethanol consumed (ml; converted to g/kg) were recorded for analysis.

Experiments were conducted using a within-subject design, with 3–4 days washout between administration of different doses, which were counterbalanced using a Latin square design. One group of *n* = 10 rats was used to test the effects of SB334867, LSN2424100, and almorexant on operant responding maintained on a progressive ratio schedule, in separate experiments. Drugs were administered two days per week (Tues and Fri) to allow for washout between subsequent doses. Rats received vehicle, 3, 10, or 30 mg/kg SB334867 (i.p., 30 min prior to the session); vehicle, 3, 10, or 30 mg/kg LSN2424100 (i.p., 30 min prior to the session); or vehicle, 10, 30, or 60 mg/kg almorexant or 60 mg/kg of the inactive enantiomer of almorexant (p.o., 60 min prior to the session). On all other days, rats received progressive ratio operant testing without any drug treatments to maintain operant performance and confirm return to baseline behaviors. One rat was excluded from testing 60 mg/kg almorexant due to observation of a skin rash not related to the study drug.

#### Binge drinking in C57BL/6J mice

One week prior to ethanol intake testing, mice were given daily saline injections (i.p.) to acclimate them to handling and injection procedures. Ethanol consumption was assessed using a 4-day drinking-in-the-dark (DID) paradigm during which the water bottle in the home cage was replaced with a single bottle of ethanol (20% v/v) starting 3 h after the onset of the dark cycle. This procedure has been shown to produce high blood ethanol concentrations (BECs) resulting from high levels of ethanol consumption in a relatively short period of time (Rhodes et al., 2005). On the first three days, animals were injected with saline or vehicle 30 min prior to a 2-h period of access to ethanol. On the 4th day, drugs were administered via i.p. injection 30 min prior to the test session, which was extended to 4 h. One cohort of mice was administered vehicle, 3, 10, or 30 mg/kg SB334867 (*n* = 10/dose). A second cohort of mice was tested with vehicle, 15, 30, or 60 mg/kg LSN2424100 (*n* = 9–10/dose). A third cohort of animals was given vehicle, 25, 50, or 100 mg/kg almorexant (*n* = 10/dose). In order to assess resulting BECs, immediately upon removal of ethanol bottles, blood samples were collected from the retro-orbital sinus and centrifuged. The plasma was assayed using an Analox Instruments analyzer (Lunenburg, MA).

In order to assess the specificity of drug effects on ethanol consumption, an additional group of ethanol-naïve animals was tested with sucrose solution (1% w/v) in the same DID paradigm (Days 1–3: 2-h access with saline injections; Day 4: 4-h test session with drug pretreatment). On the 4th day, vehicle, 3, 10, or 30 mg/kg (*n* = 6–7/dose) SB334867 was administered prior to the 4-h access period. During a subsequent week of testing, these same mice were administered either vehicle or 100 mg/kg almorexant (*n* = 14/dose) before the 4-h intake session. In a separate cohort of mice, vehicle or 60 mg/kg LSN2424100 (*n* = 9–10/dose) was administered prior to the 4-h test.

#### Data analysis

For subjects treated with SB334867 and LSN2424100 in the home cage drinking studies, ethanol and water intake (g/kg and ml/kg, respectively) during the first 3 h of the dark cycle were calculated and analyzed separately using repeated measures analyses of variance (ANOVAs), with drug dose as a within-subjects factor (IBM SPSS Statistics, Armonk, NY). The positive control, naltrexone, was compared with vehicle using an independent samples *t*-test. For subjects treated with almorexant, ethanol and water intake were analyzed using ANOVAs with drug dose as a between-subjects factor. The positive control, naltrexone, was compared with vehicle using an independent samples *t*-test. All significant within-subject effects were further explored using paired-samples *t*-tests, except for the between-subjects almorexant study, for which Tukey's HSD test was used.

For subjects treated with SB334867 and LSN2424100 in the operant progressive ratio paradigm, total active lever responses, total ethanol consumption (g/kg), and breakpoints (highest FR value reached) were calculated and analyzed via repeated measures ANOVAs, with dose as a within-subjects factor. The negative control, R-almorexant, was compared with vehicle using a separate paired samples *t*-test. All significant within-subject effects were further explored using paired-samples *t*-tests. Total responding on the inactive levers was negligible, so those data were not analyzed.

For the mouse drinking studies, ethanol consumption during each 4-h test session was expressed in g/kg and subjected to a One-Way ANOVA with dose as a between-subjects factor. BEC data (mg/dl) were analyzed similarly. Sucrose data were expressed as ml/kg and subjected to independent samples *t*-tests. Because only the doses of LSN2424100 and almorexant that reduced ethanol consumption were tested with sucrose, the 3 and 10 mg/kg SB334867 data were excluded from the analysis for consistency across compounds.

## Results

### Home cage 2-bottle choice drinking in P rats

#### SB334867

**Figure 1:**
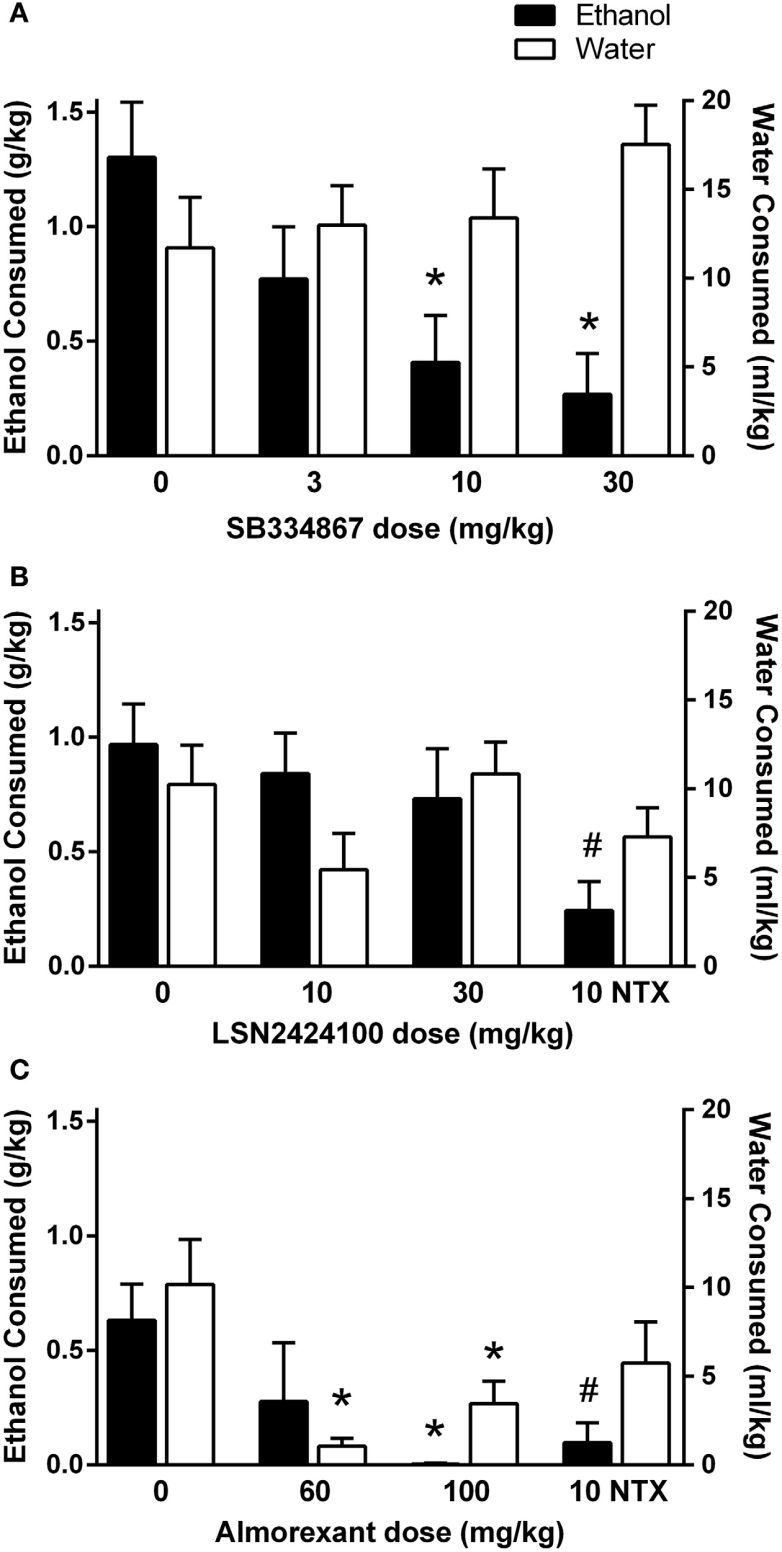
**Home cage 2-bottle choice drinking in P rats during the first 3 h of the dark cycle. (A)** SB334867 (*n* = 10) reduced ethanol intake at doses of 10 and 30 mg/kg, without altering water consumption. **(B)** LSN2424100 (*n* = 10) did not significantly influence ethanol or water intake. Naltrexone was included as a positive control at a dose (10 mg/kg) that selectively reduced ethanol intake (indicated by #, *p* < 0.05). **(C)** Almorexant (*n* = 8/dose) reduced ethanol intake at the 100 mg/kg dose while also suppressing water intake at the 60 and 100 mg/kg doses. Naltrexone was included as a positive control at a dose (10 mg/kg) that selectively reduced ethanol intake (indicated by #, *p* < 0.05). Filled bars indicate ethanol consumption (g/kg) on the left y-axis; open bars indicate water consumption (ml/kg) on the right y-axis. ∗ indicates significant difference (*p* < 0.05) relative to vehicle-treated controls.

In the first experiment, rats received vehicle, 3, 10, or 30 mg/kg SB334867. A significant main effect of dose was observed [*F*_(3, 27)_ = 4.36, *p* < 0.05]. Follow-up paired-samples *t*-tests indicated that the 10 and 30 mg/kg doses of SB334867 significantly reduced ethanol intake relative to vehicle (*p*s < 0.05 and 0.01, respectively; Figure 1A, filled bars). No significant effects emerged in the analysis of water consumption (*p* > 0.05; Figure 1A, open bars).

#### LSN2424100

In the second experiment, rats received vehicle, 10, or 30 mg/kg LSN2424100, or 10 mg/kg naltrexone. As shown in Figure 1B, LSN2424100 did not significantly affect ethanol intake (*p* > 0.05; filled bars). However, a paired-samples *t*-test revealed that naltrexone significantly attenuated ethanol intake [*t*_(9)_ = 3.31, *p* < 0.015]. Neither LSN2424100 nor naltrexone produced significant effects on water intake (*p*s > 0.05; Figure 1B, open bars).

#### Almorexant

In the third experiment, rats received vehicle, 60 or 100 mg/kg almorexant, or 10 mg/kg naltrexone in a between-subjects design. Almorexant significantly reduced ethanol intake [*F*_(2, 20)_ = 3.12, *p* = 0.05]. A *post-hoc* Tukey's HSD test revealed that only the 100 mg/kg dose of almorexant significantly affected ethanol intake, while effects at the 60 mg/kg dose were not statistically significant (Figure 1C, filled bars). An independent-samples *t*-test revealed that naltrexone also significantly reduced ethanol consumption [*t*_(13)_ = 3.08, *p* < 0.01; Figure 1C, filled bars]. Almorexant significantly reduced home cage water intake [*F*_(2, 21)_ = 10.60, *p* < 0.01; Figure 1C, open bars]. A *post-hoc* Tukey's HSD test revealed that both the 60 and 100 mg/kg doses of almorexant significantly reduced water consumption (*p*s < 0.01).

### Operant progressive ratio responding in P rats

#### SB334867

**Figure 2:**
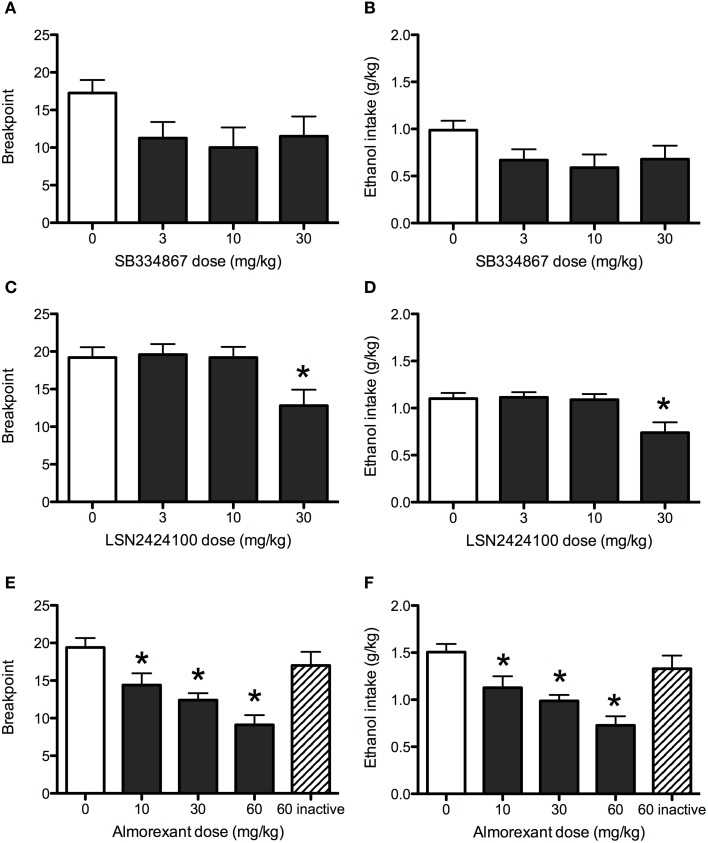
**Operant progressive ratio responding in P rats.** SB334867 (*n* = 8) did not significantly reduce breakpoints **(A)** or ethanol consumption **(B)** maintained by a progressive ratio operant schedule of reinforcement. The 30 mg/kg dose of LSN2424100 (*n* = 10) reduced the motivation to consume ethanol as indicated by reductions in breakpoints **(C)** and corresponding ethanol consumption **(D)**. **(E,F)** Almorexant (*n* = 10) reduced breakpoints and ethanol consumption at all doses tested (10, 30, and 60 mg/kg) doses. As expected, the inactive enantiomer (60 mg/kg) did not significantly affect progressive ratio operant responding for ethanol or ethanol consumption. Breakpoint was defined as the highest fixed ratio value reached by rats during the operant session. ∗ indicates significant difference (*p* < 0.05) relative to vehicle-treated controls.

Although a trend toward efficacy was observed for SB334867 on progressive ratio responding for ethanol, statistical analysis revealed no significant effects of the drug on the number of active lever responses (data not shown), breakpoints (Figure 2A), or total ethanol consumption (Figure 2B; all *p*s > 0.05).

#### LSN2424100

The OX_2_ antagonist LSN2424100 significantly reduced breakpoints [main effect of dose: *F*_(3, 36)_ = 4.61, *p* < 0.01] and resulting ethanol consumption [main effect of dose: *F*_(3, 36)_ = 6.59, *p* < 0.01]. *Post-hoc* tests indicated that the 30 mg/kg dose was significantly different from vehicle (see Figures 2C,D). LSN2424100 also significantly reduced active lever presses [*F*_(3, 27)_ = 3.67, *p* < 0.05; data not shown].

#### Almorexant

Almorexant significantly reduced active lever presses [main effect of dose: *F*_(3, 24)_ = 25.26, *p* < 0.001]. Paired-samples *t*-tests indicated that doses of 10, 30, and 60 mg/kg significantly reduced active lever responding (*p*s < 0.05; data not shown). A paired-samples *t*-test indicated that the inactive enantiomer of almorexant did not significantly affect responses on the active lever (*p* > 0.05). Similarly, almorexant significantly reduced breakpoints [*F*_(3, 24)_ = 32.32, *p* < 0.001], with significant effects at doses of 10 (*p* < 0.01), 30 and 60 mg/kg (*p*s < 0.001; Figure 2E), while the inactive enantiomer did not (*p* > 0.05). Almorexant also significantly attenuated ethanol consumption [*F*_(3, 24)_ = 32.29, *p* < 0.001] at doses of 10 (*p* < 0.01), 30, and 60 mg/kg (*p*s < 0.001; Figure 2F). A paired-samples *t*-test indicated that the inactive R-enantiomer of almorexant did not significantly affect ethanol consumption (*p* > 0.05).

### Binge drinking in C57BL/6J mice

#### SB334867

**Figure 3:**
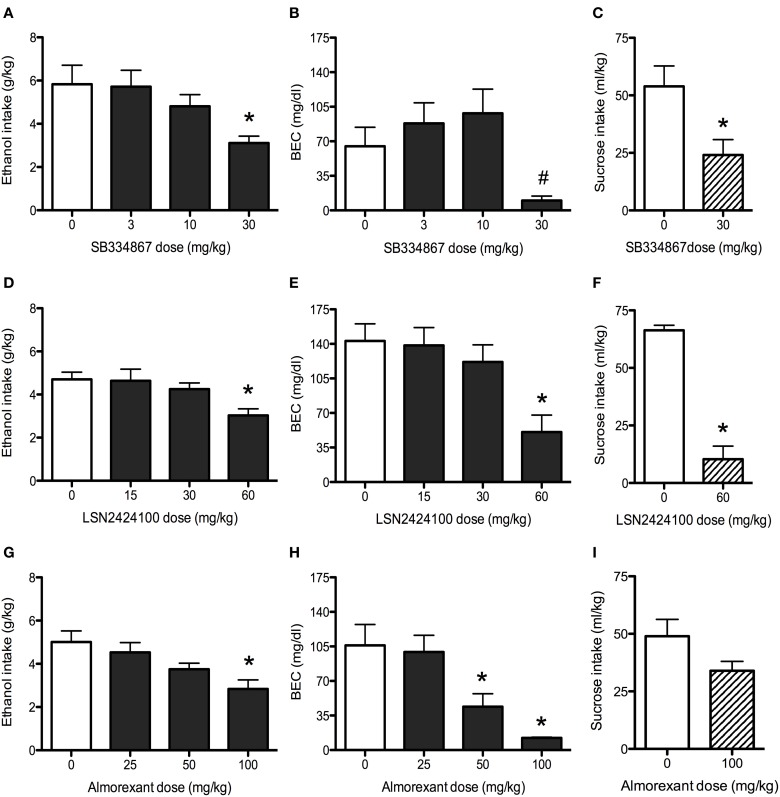
**Binge drinking in C57BL/6J mice. (A)** SB334867 (*n* = 10) reduced ethanol consumption at the 30 mg/kg dose. **(B)** The 30 mg/kg dose of SB334867 resulted in lower BECs relative to both the 10 and 30 mg/kg doses (indicated by #, *p* < 0.05). **(C)** In a separate cohort of mice, SB334867 (30 mg/kg) also reduced consumption of a 1% sucrose solution (*n* = 6–7). **(D)** LSN2424100 (*n* = 10) reduced ethanol consumption at the 60 mg/kg dose. **(E)** The same dose resulted in lower BECs relative to vehicle-injected controls. **(F)** The 60 mg/kg dose of LSN2424100 significantly reduced consumption of a 1% sucrose solution (*n* = 9–10). **(G)** Almorexant (*n* = 10) reduced ethanol intake at the 100 mg/kg dose. **(H)** Both the 50 and 100 mg/kg doses of almorexant resulted in lower BECs. **(I)** In a separate cohort of mice, almorexant (100 mg/kg) did not significantly reduce consumption of a 1% sucrose solution (*n* = 14). ∗ indicates significant difference (*p* < 0.05) relative to vehicle-injected controls.

Analysis revealed a main effect of dose [*F*_(3, 36)_ = 3.6, *p* < 0.05], with the 30 mg/kg SB334867 dose reducing ethanol intake relative to vehicle-injected controls (Figure 3A). BEC data indicated a similar pattern, with a main effect of dose emerging [*F*_(3, 36)_ = 4.4, *p* < 0.05]. While *post-hoc* tests revealed no significant differences between vehicle and any doses of SB334867, pairwise comparisons indicated that the 30 mg/kg dose resulted in lower BECs than both the 3 and 10 mg/kg doses (Figure 3B). Analysis of sucrose consumption (Figure 3C) revealed that 30 mg/kg SB334867 suppressed sucrose intake relative to vehicle [*t*_(11)_ = 2.74, *p* < 0.05].

#### LSN2424100

Analysis of ethanol intake on the test day revealed a main effect of dose [*F*_(3, 35)_ = 4.3, *p* < 0.05], with the 60 mg/kg dose reducing ethanol consumption relative to vehicle-injected control mice (Figure 3D). This reduction was mirrored in BEC data [main effect of dose: *F*_(3, 35)_ = 6.1, *p* < 0.01], with the 60 mg/kg dose resulting in lower BECs (Figure 3E). Analysis of sucrose consumption data (Figure 3F) revealed a significant suppression in mice administered the 60 mg/kg dose relative to vehicle [*t*_(17)_ = 8.76, *p* < 0.001].

#### Almorexant

Analysis revealed a main effect of dose [*F*_(3, 36)_ = 5.0, *p* < 0.01], with the 100 mg/kg dose reducing ethanol intake relative to vehicle-injected controls (Figure 3G). Analysis of BEC data also revealed a main effect of dose [*F*_(3, 36)_ = 9.0, *p* < 0.001], with both the 50 and 100 mg/kg doses of almorexant resulting in lower BECs than vehicle-treated control mice (Figure 3H). Analysis of sucrose consumption data revealed a trend (*p* = 0.085) for almorexant to reduce sucrose consumption, an effect that did not reach statistical significance (Figure 3I).

## Discussion

Data from the present series of experiments provide evidence that blockade of OX_1_ and OX_2_ receptors reduces ethanol self-administration in a variety of high-drinking rodent paradigms, although observed effects were dependent on the specific procedures used to evaluate ethanol-seeking behavior. The OX_1_ receptor antagonist reduced home cage ethanol drinking in rats and binge-like drinking in mice, without influencing progressive ratio operant responding in rats. Blockade of OX_2_ receptors did not alter home-cage ethanol intake in rats, but did lower breakpoints and reduce ethanol consumption in the progressive ratio procedure in P rats as well as reducing binge-like drinking in mice. Dual antagonism of OX_1_ and OX_2_ receptors resulted in reduced ethanol consumption in rats and mice in addition to decreasing breakpoints and ethanol consumption in the operant progressive ratio model. Due to an established role for orexin in the regulation of feeding behavior, it was important to assess the specificity of these drug effects by measuring the ability of the these compounds to alter consumption of another caloric solution. Indeed, results from the present study indicated that some of the test compounds also reduced sucrose consumption in mice.

The majority of previous work exploring the role of orexin in ethanol reward has focused on blockade of OX_1_ receptors with SB334867, with this compound typically reducing ethanol self-administration (Lawrence et al., 2006; Richards et al., 2008; Moorman and Aston-Jones, 2009; Jupp et al., 2011). In accordance with these findings, we demonstrate here that SB334867 reduced home cage ethanol intake in P rats with a long history of ethanol consumption. SB334867 has been previously shown to reduce breakpoints in an operant progressive ratio procedure in male iP rats (Jupp et al., 2011). In contrast, SB334867 did not significantly alter breakpoints or corresponding ethanol consumption in female P rats in the present study. It is unclear whether this discrepancy can be attributed to sex differences, experimental procedural differences, or differences between respective inbred lines. In the current study, we reported for the first time that SB334867 reduced binge-like ethanol intake in the drinking-in-the-dark procedure in mice. Oddly, the BEC values reported in the vehicle-injected mice were lower than expected given the high ethanol consumption. This apparent disparity may be a consequence of the long length of the testing period. Although not assessed in the current study, different patterns of ethanol consumption over the 4-h period may have resulted in different BEC values. The dose of SB334867 that effectively reduced ethanol intake also suppressed consumption of a 1% sucrose solution. However, previous reports indicate that SB334867 did not affect operant self-administration of a 5% sucrose solution (Richards et al., 2008). It is tempting to speculate that self-administration of the 1% sucrose solution used in the current experiments may be more susceptible to disruption by OX antagonists because it is less palatable to the mice than the 5% sucrose solution in other studies. Further experiments will be required to explore this possibility. Overall, the data reported herein complement and extend previous literature reports demonstrating that SB334867 attenuates ethanol self-administration.

Although previous work has examined OX_2_ receptor involvement in operant ethanol self-administration and reinstatement procedures, the present study is the first to examine the effects of OX_2_ antagonism on voluntary home cage ethanol consumption, breakpoints in an operant progressive ratio procedure, and binge-like ethanol drinking using the novel compound LSN2424100. This OX_2_ antagonist did not alter ethanol consumption under voluntary continuous access conditions in P rats but did reduce breakpoints in the operant progressive ratio procedure, indicating reduced motivation to consume ethanol (Richardson and Roberts, 1996) in alcohol-preferring rats. Consistent with the reduction in operant breakpoints, LSN2424100 also decreased ethanol intake in the progressive ratio model. Our data are consistent with and complement earlier studies in which other selective OX_2_ receptor antagonists (JNJ-10397049 and TCS-OX2-29) decreased ethanol self-administration using other operant paradigms in rats (Shoblock et al., 2011; Brown et al., 2013).

Because it is well-known that OX antagonists reduce wakefulness and suppress motor activity in general (e.g., Brisbare-Roch et al., 2007), one might argue that the breakpoint reduction seen in P rats is simply due to reduced locomotor activity. This is unlikely given that the same dose of LSN2424100 (30 mg/kg) did not alter ethanol or water consumption in the home cage drinking procedure reported here. Indeed, although OX receptor antagonists facilitate sleep, they do not produce overt motor impairment or sedative-like effects that are commonly associated with benzodiazepine receptor agonists, such as zolpidem (Steiner et al., 2011). To the contrary, rats receiving OX receptor antagonists, including those tested here, can perform operant and other motor tasks without any observable impairment (Steiner et al., 2011; Rorick-Kehn et al., unpublished observations). That LSN2424100 reduced ethanol self-administration in the operant progressive ratio assay, but not when ethanol was provided under unlimited access conditions, may suggest that OX_2_ receptor-mediated signaling does not directly modulate ethanol reward. Rather, OX_2_ receptors may be involved in modulating motivational circuits in the brain that underlie drug-seeking behavior. Indeed, it is not uncommon for drugs to differentially influence ethanol's appetitive/motivational effects vs. consummatory behavior (ethanol drinking *per se*) (e.g., Czachowski et al., 2001, 2002). Alternatively, the differential efficacy of LSN2424100 in these two procedures may reflect the varying ethanol histories of the rats tested in each model.

In mice, a high dose of LSN2424100 (60 mg/kg) reduced ethanol consumption in the binge-like drinking procedure; however, the same dose also suppressed sucrose consumption, suggesting that this dose was high enough to produce non-specific effects. Whether the suppression in sucrose intake reflects sleep-promoting effects or a general reduction in consummatory behaviors cannot be determined from the present series of studies. It is interesting that the reduction in sucrose intake (~85% reduction) was more dramatic than the reduction in ethanol intake (~36% reduction). The reasons for this difference are not clear. Further work will be necessary to better characterize the nature of these effects.

Antagonism of both OX_1_ and OX_2_ receptors by almorexant reduced ethanol drinking in rats with continuous home-cage access and in limited access binge-drinking in mice, and also attenuated breakpoints and ethanol consumption in the progressive ratio model in rats. The data presented here confirm and extend previous reports that almorexant suppressed operant self-administration of both ethanol and sucrose in Long-Evans rats (Srinivasan et al., 2012). Importantly, we demonstrate here that the inactive enantiomer of almorexant did not suppress breakpoints or ethanol self-administration in rats, indicating that the effect was specific to blockade of OX_1_ and OX_2_ receptors rather than unknown off-target pharmacological effects. Our data in regard to the specificity of almorexant effects were mixed. Specifically, almorexant reduced home-cage water drinking in rats, suggesting potential non-specific effects on fluid consumption, but it did not significantly attenuate sucrose intake in the mice. Almorexant has previously been shown to reduce operant responding for both ethanol and 5% sucrose when administered systemically; however, when administered directly into the VTA, effects were selective for ethanol (Srinivasan et al., 2012). In the current report, it is unclear whether the reduced water intake in rats, and the tendency for reduced sucrose intake in mice, reflects non-specific consummatory effects or transient sedative effects that may dissipate over the course of the extended drinking session. Additional examination of the selectivity of effects of the compounds tested in the present work, including assays of locomotor activity, is warranted.

The drinking paradigms employed in the present study involved different amounts of total ethanol exposure. Previous studies have reported alterations in the orexin system following long-term ethanol exposure. For example, chronic voluntary ethanol consumption (~5 g/kg/day for 70 days) has been reported to upregulate hypothalamic preproorexin mRNA in P rats (Lawrence et al., 2006) whereas a reduction in orexin mRNA has been reported after chronic ethanol consumption (~0.75–2.5 g/kg/day for 28 days) in Sprague-Dawley rats (Morganstern et al., 2010). Several methodological differences between the two studies may account for the seemingly contradictory results, including differences in total daily ethanol exposure (~5 g/kg/day vs. ~0.75–2.5 g/kg/day), differences in genetic background (selectively bred P rats vs. Sprague-Dawley rats), and endpoint measured (preproorexin vs. orexin A mRNA). Nonetheless, further studies will be required to determine the impact of long-term ethanol exposure on the brain orexin system, and whether adaptations in orexin signaling resulting from chronic ethanol exposure contribute to the development of addiction. Indeed, others have demonstrated that orexin-A stimulates dopamine cell firing in the VTA, increases dopamine release in the prefrontal cortex (PFC), and potentiates PFC-evoked excitation of VTA dopamine cells (Narita et al., 2006; Vittoz and Berridge, 2006; Moorman and Aston-Jones, 2010). Moreover, Borgland et al. (2006) demonstrated that orexin signaling in the VTA plays a critical role in synaptic plasticity associated with cocaine addiction. The relevance of orexin-mediated signaling in critical processes associated with addiction to drugs of abuse, including ethanol, is beginning to be understood, and will likely be further informed by the development and characterization of additional selective tool compounds from different chemical scaffolds, such as LSN2424100 reported here, that can be used to explore the relative roles of OX_1_- and OX_2_-receptor-mediated signaling. Future studies in models of ethanol dependence using selective pharmacological tools may provide valuable information about the therapeutic potential of orexin antagonists for the treatment of alcohol abuse and alcoholism.

### Conflict of interest statement

Financial support for P-rat studies was provided by Eli Lilly and Company. Benjamin L. Adams, Cynthia D. Jesudason, and Linda M. Rorick-Kehn are employees of, and stockholders in, Eli Lilly and Company.
